# Abrin Immunotoxin: Targeted Cytotoxicity and Intracellular Trafficking Pathway

**DOI:** 10.1371/journal.pone.0058304

**Published:** 2013-03-05

**Authors:** Sudarshan Gadadhar, Anjali A. Karande

**Affiliations:** 1 Department of Biochemistry, Indian Institute of Science, Bangalore, India; Wayne State University, United States of America

## Abstract

**Background:**

Immunotherapy is fast emerging as one of the leading modes of treatment of cancer, in combination with chemotherapy and radiation. Use of immunotoxins, proteins bearing a cell-surface receptor-specific antibody conjugated to a toxin, enhances the efficacy of cancer treatment. The toxin Abrin, isolated from the *Abrus precatorius* plant, is a type II ribosome inactivating protein, has a catalytic efficiency higher than any other toxin belonging to this class of proteins but has not been exploited much for use in targeted therapy.

**Methods:**

Protein synthesis assay using ^3^[H] L-leucine incorporation; construction and purification of immunotoxin; study of cell death using flow cytometry; confocal scanning microscopy and sub-cellular fractionation with immunoblot analysis of localization of proteins.

**Results:**

We used the recombinant A chain of abrin to conjugate to antibodies raised against the human gonadotropin releasing hormone receptor. The conjugate inhibited protein synthesis and also induced cell death specifically in cells expressing the receptor. The conjugate exhibited differences in the kinetics of inhibition of protein synthesis, in comparison to abrin, and this was attributed to differences in internalization and trafficking of the conjugate within the cells. Moreover, observations of sequestration of the A chain into the nucleus of cells treated with abrin but not in cells treated with the conjugate reveal a novel pathway for the movement of the conjugate in the cells.

**Conclusions:**

This is one of the first reports on nuclear localization of abrin, a type II RIP. The immunotoxin mAb F1G4-rABRa-A, generated in our laboratory, inhibits protein synthesis specifically on cells expressing the gonadotropin releasing hormone receptor and the pathway of internalization of the protein is distinct from that seen for abrin.

## Introduction

Chemotherapy is the most common modes of treatment of cancer. However, its success and efficacy are challenged because of the side effects associated with the treatment, majorly caused due to the inhibition also of fast proliferating normal cells of the body. Use of other modalities of treatment to combat cancer is the need of the hour and of late monoclonal antibodies (mAbs) are one of the front runners as potential drugs for treating cancer. Apart from their use in antibody mediated cell and complement-mediated cytotoxicity, mAbs can be linked to various anti-cancer drugs, radionuclides and toxins [1]–[3]. This not only ensures site-specific delivery of the therapeutic molecules but also maximizes the effect of the drug and minimizes side effects [1], [3], [4]. In several cancer cells, there is up-regulation of tumor associated antigens and specific cell-surface receptors, which can be targeted with ‘immunotoxins’. The toxins used in synthesizing these conjugates can be ribosome inactivating proteins (RIPs), those that specifically inhibit the eukaryotic ribosome, leading to inhibition of protein synthesis, following which cells undergo programmed cell death [5]–[9]. Hence RIPs are potent weapon candidates for use in immunotherapy of various diseases, including cancer [5], [10].

Immunotoxins can be defined as conjugates of a toxin with an antibody, the whole molecule or only the antigen binding regions: the Fv or Fab. Immunotoxins can also be ‘recombinant or fusion toxins’ when the genes for both the antibody and the toxin are ligated, cloned into bacterial system and expressed as fusion proteins [11], [12]. Immunotoxins reported till now have been constructed using the toxins saporin, mistletoe lectin-1, gelonin, pokeweed antiviral protein (PAP) and ricin from plant sources and shiga toxin, diphtheria toxin and *Pseudomonas* exotoxin from bacterial sources [12]–[14], either using the holotoxin or the purified A chain of ricin [15]. Apart from ricin, other more potent toxins that can be considered for immunotoxin construction are volkensin [16], stenodactylin [17] and abrin [18], whose toxicity is much higher when compared to ricin. Abrin, isolated from the plant *Abrus precatorius* is a type II RIP, has an enzymatic A chain having RNA-N-glycosidase activity, linked by a single disulfide linkage to the B chain, a lectin with specificity to terminal galactose [5], [19]. Abrin has a lower Km than any other type II RIPs [18], [20] and also the maximum catalytic efficiency in that one molecule can inhibit approximately 2000 ribosomes/min [21].

Utilizing holotoxins [3]–[5] has the drawback of non-specific binding of the immunoconjugate to all cells via the B chain [22], [23]. Therefore, we proposed to use the recombinant abrin-a A chain (rABRa-A) expressed in *E. coli*. As immunotoxin should kill cancer cells preferentially, expression of the target molecules should be higher on cancer cells as compared to the normal ones. Expression of gonadotropin releasing hormone receptor (GnRH-R) on breast carcinoma cells is reported to be higher than those on the normal breast tissue [24]–[26]. Therefore we proposed to utilize MCF-7 (breast carcinoma cell line) and MCF-10A (transformed, non-cancerous breast cell line) as model partners for our studies [27], [28]. To study the absolute specificity of the conjugate, a liver cell line, HepG2 that overexpresses GnRH-R was then recruited. GnRH-R have not been targeted extensively, with only a few reports of gelonin and PAP based immunotoxins targeting the receptor [29], [30]. Hence, our study aimed at determining the possibility of using GnRH-R as a potent target for immunotherapy.

Type II RIPs have a well-established trafficking pathway [31]–[35], involving the B chain for binding to the galactose. This is followed by internalization and retrograde transport pathway to the endoplasmic reticulum (ER). The A chain is released from the ER through the ER associated degradation (ERAD) pathway [35]. However, the trafficking of an immunoconjugate within the cell would be receptor-dependent and might differ from cell to cell. Hence, we analyzed a few steps of the movement of the immunoconjugate bound to the GnRH-R to understand the pathway of trafficking of the protein.

## Materials and Methods

### Cells

The human cell lines, MCF-7 (breast carcinoma), HepG2 (hepatocarcinoma), KB (nasopharyngeal carcinoma) [36] were procured from the Cell Repository of the National Centre for Cell Science, Pune, India and MCF-10A (human normal breast cell line) from Prof. Annapoorni Rangarajan, MRDG, Indian Institute of Science, Bangalore, India [37]. MCF-7, HepG2 and KB cells were maintained in Dulbecco’s Modified Eagle’s Medium (DMEM), supplemented with 10% fetal bovine serum and 2 mM Glutamax (Invitrogen Corporation, USA) at 37°C in a humidified 5% CO_2_ incubator. MCF-10A cells were maintained in DMEM-Ham’s F12 (Sigma-Aldrich Co.) supplemented with 10% fetal bovine serum, 20 ng/ml epidermal growth factor, 10 µg/ml insulin, 0.5 µg/ml hydrocortisone and 2 mM Glutamax. The adherent cultures were grown as monolayer and were passaged once in 4–5 days by trypsinizing.

### Antibodies for Immunotoxin

MAbs F1G4 and A9E4 [38] were raised against a peptide of the extracellular domain of the GnRH-R of which, mAb F1G4 has been shown to bind to the receptor. MAb VU1D9 [39] is an epithelial cell adhesion molecule (EpCAM) specific antibody.

### Immunofluorescence

MCF-7, HepG2, MCF-10A and KB cells (4×10^4^ cells/mm^2^), plated on cover slips, were fixed with 4% paraformaldehyde for 20 min at room temperature (RT), washed with 50 mM phosphate buffer, pH 7.4, containing 150 mM NaCl (PBS) containing 1% BSA and blocked with the same solution for 1 h at RT. Cells were incubated with the antibodies overnight at 4°C followed by incubation with FITC-conjugated rabbit anti-mouse Ig for 1 h at RT. The cells were counterstained with Hoechst 33342 (1 µg) for 5–10 min. Images were acquired using either the Olympus DSU microscope or the Apotome.2 (Carl Zeiss) and analyzed using either the Image J browser or the AxioVision Rel. 4.8.2 from Carl Zeiss.

### In-vitro Translation Assay

Clones of rABRa-A or the active site mutant, rABRa-A (R167L) were a kind gift from Prof. J.Y Lin, National Taiwan University, Taiwan, Republic of China. *E. coli* cells transformed with the plasmid were induced to express the protein as described [40]. The activity of the purified rABRa-A and rABRa-A (R167L) was determined using the *in-vitro* translation assay (Promega Pte. Ltd, Singapore) [41]. Briefly, the rabbit reticulocyte lysate was incubated with varying concentrations of rABRa-A or rABRa-A (R167L) ranging from 10 pM to 1000 pM in 0.25 µl of PBS in a reaction cocktail containing luciferase mRNA, at 37°C for 1 h. The reaction mixture was mixed with the luciferase substrate and the amount of product formed was measured in a luminometer.

### Conjugation

The immunotoxins were constructed using standard protocols [42]. The cross-linker, Succinimidyloxycarbonyl-α-methyl-α-(2-pyridyldithio)toluene [SMPT] (Thermo Scientific, Rockford, USA), in dimethyl sulfoxide (DMSO), was added to the antibody (2 mg/ml in PBS) at a final concentration of 0.13 mg/ml, mixed gently, and incubated at RT for 1 h. The unreacted SMPT was removed by desalting. The toxin, at 1 mg/ml in PBS, was degassed, incubated with 2.5 mM dithiothreitol (DTT) for a period of 1 h at RT and mixed with the activated antibody in a ratio of 2 mg antibody per mg of the toxin. After filter-sterilizing using a 0.22 µm filter, the solution was incubated under nitrogen at RT for 18 h. Excess pyridyl disulfide active sites were blocked with 25 µg/ml cysteine at RT for 6 h. To purify the conjugate from the unconjugated antibody and toxin, the mixture was chromatographed first on Cibacron blue 3GA agarose and then on protein A agarose column.

### Protein Synthesis Assay

MCF-7, HepG2, KB and MCF-10A cells: 0.2×10^6^ cells (1×10^6^ cells/ml) plated overnight were cultured in 200 µl of L-leucine free RPMI, with different concentrations of the various immunoconjugates for 8 h at 37°C. The cells were pulsed with 0.1 µCi ^3^[H] L-leucine (BRIT, India) for 2 h and the total protein was precipitated overnight using 5% (w/v) trichloro acetic acid (TCA). The precipitate was washed with ethanol, solubilized with 200 µl of 1% sodium dodecyl sulfate (SDS) in 0.1 N NaOH and the radioactivity was measured in a liquid scintillation counter [43], [44].

To determine the involvement of thioredoxin (Trx)-thioredoxin reductase (TrxR) complex in the reduction of mAb F1G4-rABRa-A, we cultured HepG2 cells with different concentrations of a selective TrxR inhibitor, auranofin (Sigma-Aldrich Co.) [45] for 6 h in complete medium following which, the cells were treated with 90% translation inhibitory concentration (IC_90_) of either abrin (51.25 pM) or mAb F1G4-rABRa-A (19.2 nM) for 6 h in RPMI minus L-leucine. The cells were then pulsed with ^3^[H] L-leucine for 2 h and processed as described above. The incorporation of ^3^[H] L-leucine was determined using the liquid scintillation counter and the percent inhibition of protein synthesis in presence and absence of auranofin was analyzed.

### Induction of Cell Death in Cells by mAb F1G4-rABRa-A

HepG2 cells (0.5×10^6^) were treated with 19.2 nM (IC_90_) of the conjugates for different time intervals. The cells were then harvested, fixed with 70% ethanol treated with propidium iodide (PI) staining solution (20 µg/ml propidium iodide and 50 µg/ml RNaseA in PBS) for 1 h at 42°C. The cells were analyzed for the percentage of dead cells using flow cytometry (FACSCanto, Beckton Dickenson) [46].

### Confocal Microscopy of HepG2 Cells to Analyze the Trafficking of the A Chain

HepG2 cells (4×10^4^/mm^2^) plated on cover slips overnight, were treated with either abrin or mAb F1G4-rABRa-A for different time intervals at 37°C. After washing off the conjugates, the cells were fixed with 4% para-formaldehyde for 10 min at RT and stained with abrin A chain specific antibody, mAb D6F10-Alexa 488, for 2 h at RT in dark followed by counterstaining with Hoechst 33342 for 10 min. The excess stain was washed off and the cover slips were mounted in presence of an anti-fade and images were acquired using the LSM 510 Meta confocal microscope (Zeiss). The images were analysed using the LSM image browser (Zeiss) [47].

### Immunoblot Analysis of Cell Lysates

Cells (5×10^6^ per 90 mm petri dish) were treated with either 6 nM abrin or 50 nM mAb F1G4-rABRa-A for different time intervals. Cells were harvested by trypsinizing, washed and re-suspended in 250 µl of homogenization buffer (0.25 M Sucrose, 10 mM HEPES, pH 7.4, 10 mM MgCl_2_, 10 mM KCl, 0.5 mM DTT, 0.1% Triton X-100 and 1 mM Phenylmethanesulfonyl fluoride (PMSF)). The cells were lysed by plunging the suspension through a syringe for 5 min on ice, incubated for 15 min and then centrifuged at 228×g for 5 min at 4°C to pellet the nuclei and other cell debris. The supernatant obtained was centrifuged at 100 000×g for 90 min at 4°C to separate the cytosol and the organellar fractions. The pellet obtained was re-suspended in 500 µl of homogenizing buffer and layered on a solution of 0.8 M sucrose containing 0.5 mM MgCl_2_ and centrifuged at 2800×g for 10 min at 4°C to get a clear nuclear pellet free of cell debris. Equal concentrations of protein was electrophoresed on 12.5% polyacrylamide gel under reducing conditions and subjected to immunoblot analysis using abrin A chain specific antibody, mAb D6F10 [48].

## Results

### MCF-7 and HepG2 Cells Express GnRH-R

The binding of all the antibodies to all the cells was analyzed by fluorescence microscopy. As expected, mAb VU1D9, an EpCAM specific antibody, bound to MCF-7, HepG2 and KB cells whereas the binding to MCF-10A cells was low as the level of EpCAM in these cells has been reported to be low as compared to its cancerous counterpart, MCF-7 cells [49]. MAb F1G4 bound to MCF-7 cells and HepG2 cells, whereas mAb A9E4 exhibited little or no binding (Fig. S1, panels A & B). Though mAb A9E4 was raised to the same GnRH-R peptide, it does not bind to the receptor [38], therefore served as an isotype control. The specificity of the binding of mAb F1G4 to the cells was confirmed by abrogation of the binding in the presence of excess of the GnRH-R peptide (Fig. S1 panel A). MAb F1G4 did not exhibit binding to KB or MCF10A cells (Fig. S1, panels C & D).

### E. coli Expressed rABRa-A is Functionally Active

The wild type rABRa-A and its active site mutant [rABRa-A (R167L)] were expressed in *E. coli* cells as 6×-His tag proteins and were purified on Ni-NTA affinity column (Fig. S2, panel A). To ascertain the activity of the recombinant A chain, an *in vitro* translation assay was carried out. The A chains were added separately to rabbit reticulocyte lysate along with the luciferase reporter mRNA. The product formed after the addition of the substrate was measured. *E. coli* expressed rABRa-A inhibited translation even at 100 pM whereas the active site mutant, rABRa-A (R167L) inhibited negligibly even at 1 nM (Fig. S2, panel B).

### Construction of Immunotoxins

The conjugation of rABRa-A was carried out with mAb VU1D9 and mAb F1G4. The conjugate was electrophoresed on a 7.5% polyacrylamide SDS gel under non-reducing conditions followed by immunoblotting with mAb D6F10. The shift in the mobility to ∼182 kDa in comparison to rABRa-A (32 kDa) and the antibody (150 kDa) confirmed the conjugation (Fig. S2, panel C).

### mAb F1G4-rABRa-A (F1G4-IT) Inhibits Protein Synthesis and is Cell-specific

**Figure 1:**
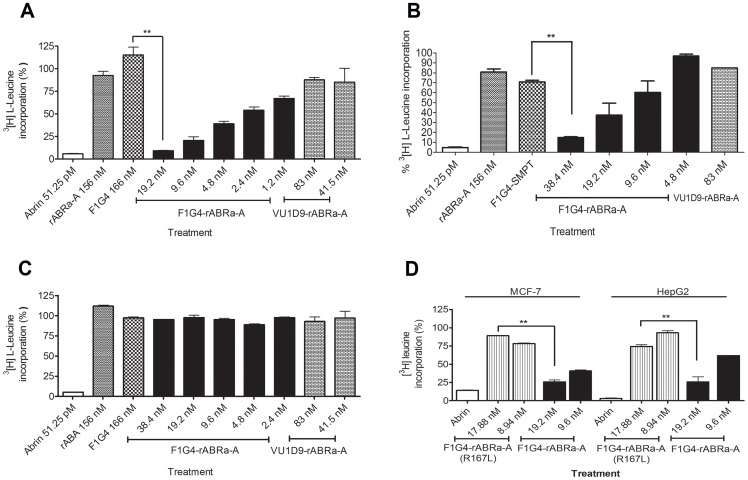
F1G4-IT inhibits protein synthesis in MCF-7 and HepG2 cells but not in KB cells. Cells were treated with F1G4-IT or mAb VU1D9-rABRa-A for 8 h in leucine free RPMI. The cells were pulsed with ^3^[H] L-leucine for 2 h and total cellular protein precipitated using 5% TCA. The incorporated radioactivity was plotted against that of the control cells. **A:** MCF-7; **B:** HepG2; **C:** KB. **D:** Cells treated with F1G4-IT or F1G4-IT**_R167L_**. Each bar represents the mean of three separate experiments carried out with duplicate samples. **P<0.05.

**Figure 2:**
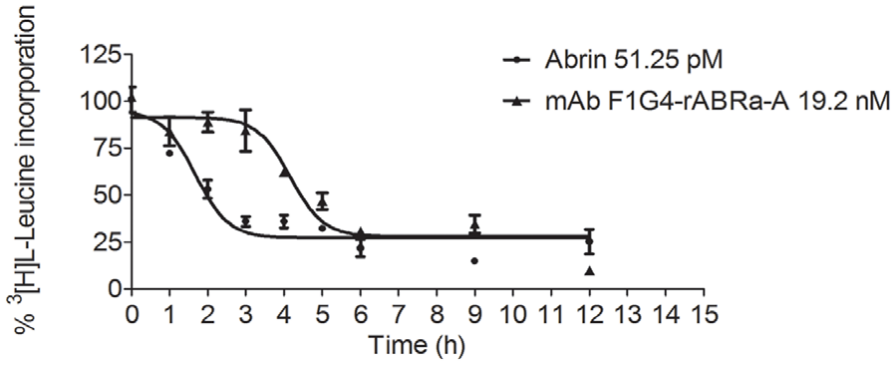
Kinetics of inhibition of protein synthesis by F1G4-IT is slower than that of abrin. HepG2 cells (1×10^6^/ml) were treated with IC_90_ of either abrin or F1G4-IT for different time intervals and the procedure followed as described in Figure 1. The Boltzmann curve was used to analyze the data. The graph represents the mean of three separate experiments carried out with duplicate samples.

- To address the ability of the immune-conjugates to inhibit protein synthesis, they were tested on MCF-7, HepG2 and KB cells. The cells were treated with different concentrations of the immunoconjugates and the extent of inhibition was compared with that by the native toxin. We observed that only F1G4-IT inhibited protein synthesis in cells bearing the GnRH-R (Fig. 1, A & B) in a dose-dependent manner and the extent was comparable to that of abrin. Of the two cell lines, MCF-7 was found to be more sensitive than HepG2 cells. As expected, neither the antibody alone, nor rABRa-A alone inhibited protein synthesis. On the other hand, no inhibition of protein synthesis was observed in KB cells (Fig. 1 C) when incubated with F1G4-IT, though abrin inhibited protein synthesis in these cells comparable to that seen in MCF-7 cells.
- To determine whether the inhibition of protein synthesis was indeed due to the active rABRa-A in the conjugate, an immunoconjugate of mAb F1G4 was constructed with the active site mutant, rABRa-A [R167L] (F1G4-IT**_R167L_**), and its activity was tested on both MCF-7 and HepG2 cells. Our results confirmed that the active rABRa-A was the inhibitory molecule, as the F1G4-IT**_R167L_** did not inhibit protein synthesis in MCF-7 cells (Fig. 1 D).
- To analyze the efficacy of F1G4-IT on normal cells in comparison to cancer cells, we determined the inhibition of protein synthesis in MCF-10A cells as well. As established by immunofluorescence microscopy, MCF-10A cells have low expression of GnRH-R (Fig. S1, panel D). Analysis of the extent of inhibition of protein synthesis by F1G4-IT in both MCF-7 and MCF-10A cells revealed that MCF-7 cells were much more sensitive to the immunoconjugate than MCF-10A cells (Fig. S3). Inhibition of protein synthesis in MCF-10A was only ∼10–15% and only with a high concentration of F1G4-IT, whereas in MCF-7 inhibition was 70% even at 9.6 nM. These results indicate that the IT is much more effective on cancer cells than normal cells.
- Since the concentration of the immunoconjugate required to inhibit protein synthesis was observed to be much higher than that of the native protein, we analyzed whether there was any difference in the kinetics of inhibition between the two molecules. HepG2 cells were treated with 90% translation inhibitory concentration (IC_90_) of either abrin or F1G4-IT for different time intervals. The kinetics of inhibition by the IT was found to be slower than that of abrin (Fig. 2). Abrin inhibited protein synthesis completely by 3 h whereas the IT required 6 h for the same effect.

### Induction of Cell Death by rABRa-A is Independent of its Protein Synthesis Inhibitory Effect

**Figure 3:**
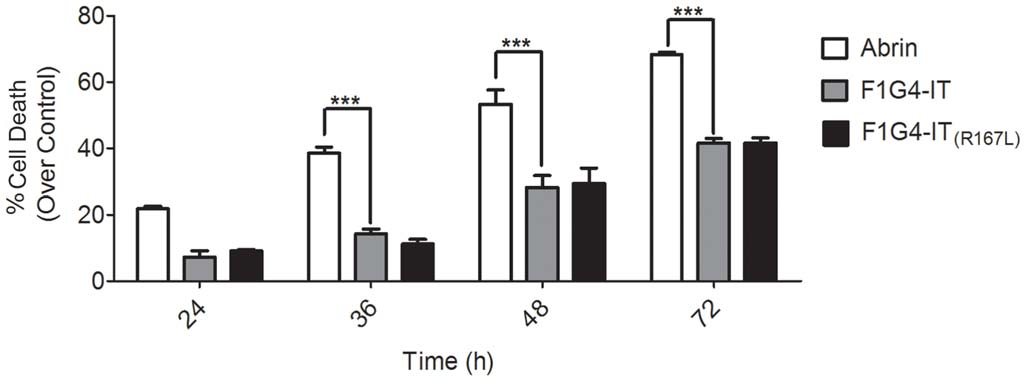
Trigger of cell death by abrin-a A chain is independent of inhibition of protein synthesis. HepG2 cells (1×10^6^/ml) were treated with 19.2 nM of either one of the immunoconjugates: F1G4-IT or F1G4-IT**_R167L_**, or abrin (51.25 pM) for different time intervals. The cells were harvested, fixed with 70% ethanol at −20°C, stained with staining solution (20 µg/ml propidium iodide and 50 µg/ml RNase A in PBS) and analyzed by flow cytometry. The percentage of dead population was determined and plotted above control cells. Each bar represents the mean of at least three different experiments carried out with duplicate samples. **P<0.005.

Though it is well-established that abrin induces cell death in cells [44], [50], [51], its link with protein synthesis inhibitory activity has been an open question. Towards unravelling this, HepG2 cells were treated with 19.2 nM of either F1G4-IT or F1G4-IT**_R167L_** for different time intervals and checked for cell death using flow cytometry. From our studies, we can infer that both the immunoconjugates were able to induce cell death in these cells to the same extent by 36 h (Fig. 3 and Fig. S4), indicating that this property of the protein is independent of inhibition of protein synthesis in HepG2 cells, as the F1G4-IT**_R167L_** fails to inhibit protein synthesis in cells. The kinetics of induction of cell death by the conjugates appears similar to that of abrin, though the extent of induction is much lesser.

### Intracellular Trafficking of mAb F1G4-rABRa-A is Different to that of Abrin

The intracellular trafficking of type II RIPs has been well established. The protein binds to the cell surface receptors and moves to the ER through the retrograde transport pathway [31]–[35], [52]. In the ER, the disulfide link between the A and the B chain of the protein is cleaved by protein disulfide isomerase and the A chain is released into the cytosol through the ERAD pathway, where it binds to its target molecule, the 60S ribosome and brings about its catalytic effect [35].

**Figure 4:**
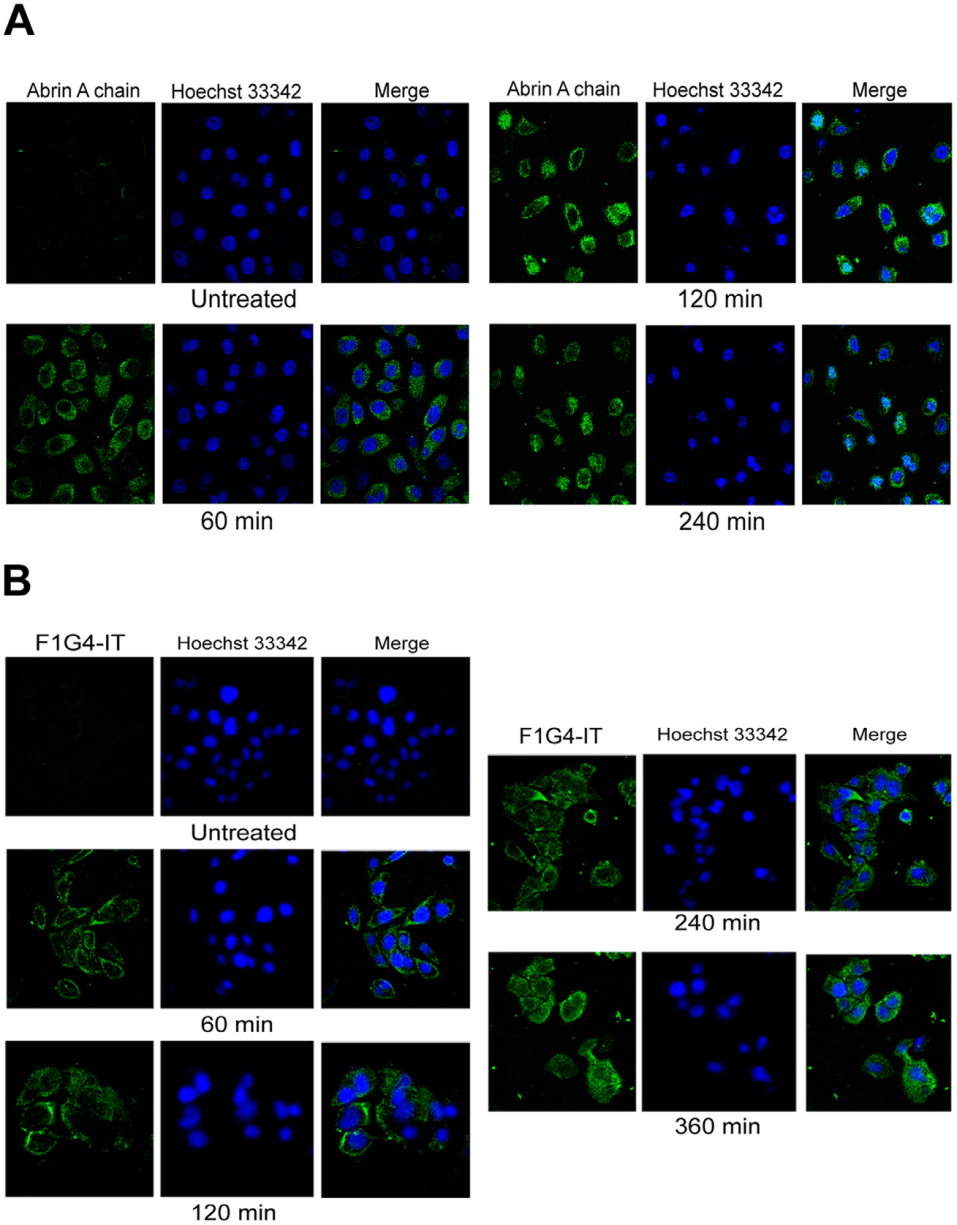
Intracellular localization of F1G4-IT in HepG2 cells is different from that of abrin. Cells (5×10^6^) treated with either abrin or F1G4-IT for different time intervals were fixed with 4% para-formaldehyde and stained with mAb D6F10-Alexa 488 for 2 h in the dark at RT. The cells were counter stained with 5 µg/ml of Hoechst 33342 for 10 min at RT, washed with PBS, mounted on slides and images acquired in the Zeiss confocal scanning microscope. The images were analysed using the Image J image browser. Confocal microscopy of, **A:** abrin treated cells; **B:** F1G4-IT treated cells.

**Figure 5:**
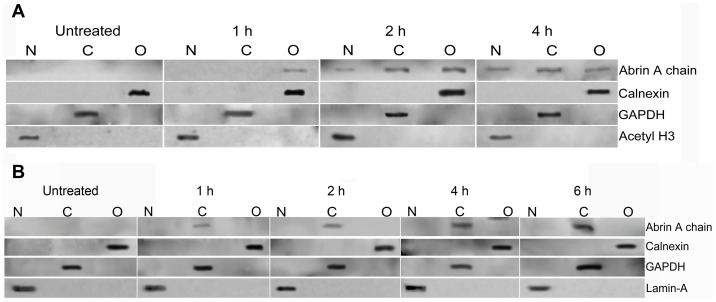
A chain of abrin and F1G4-IT have unique destinations. Cells treated with either 6 nM abrin or 50 nM F1G4-IT, for different intervals were subjected to sub-cellular fractionation. The nuclear (N), cytosolic (C) and organellar (O) fractions of each sample were electrophoresed on a 12.5% polyacrylamide SDS gel under reducing conditions and subjected to immunoblot analysis. **A:** Cells treated with abrin immunoblotted with mAb D6F10 for the A chain; Rabbit antibodies to acetylated histone, H3 (17 kDa), GAPDH (37 kDa) and Calnexin (67 kDa) were used as controls for nuclear, cytosolic and organellar fractions respectively. **B:** Cells treated with F1G4-IT immunoblotted with mAb D6F10; MAb to Lamin-A (70 kDa) and rabbit antibodies to GAPDH and Calnexin were used as controls for nuclear, cytosolic and organellar fractions respectively.

Since we found differences in the kinetics of inhibition of protein synthesis between abrin and F1G4-IT, we analyzed the intracellular localization of the immunoconjugate in comparison to abrin. HepG2 cells were treated with either 600 pM abrin or 19.2 nM F1G4-IT for different time intervals and stained with mAb D6F10-Alexa 488 to determine its localization within the cell. In cells treated with abrin, we observed localization of the A chain initially in the cytosol, but with time there was nuclear localization of the A chain (Fig. 4, panel A). We have observed that the localization of the A chain is cell-specific, wherein cells that are less sensitive to abrin toxicity, like HepG2 and KB cells, demonstrate nuclear localization of abrin and the localization is only of the A chain (unpublished observation). On the other hand, in case of cells treated with the F1G4-IT, the A chain was seen only in the cytosol of the cells, even after 6 h of treatment (Fig. 4, panel B) indicating that the protein might have a different route of travel in the cell compared to abrin. Our observations were confirmed by sub-cellular fractionation and immunoblot analysis of HepG2 cells treated with either 6 nM abrin or 50 nM F1G4-IT. The cells were fractionated into nuclei, cytoplasm and organellar fractions and were electrophoresed on a reducing 12.5% polyacrylamide SDS gel and immunoblotted with mAb D6F10. From Fig. 5, panel A, it is clear that on treatment with abrin, the A chain localized to the organelles at 60 min. There was nuclear localization of the A chain, which increased with time. Cells treated with the IT showed that the A chain was observed only in the cytosol and neither in the nucleus, and more importantly, nor the organelles at any of the time intervals (Fig. 5, panel B). Thus the F1G4-IT has a pathway of trafficking distinct from that of abrin; directly transported to the cytosol, with no movement to the ER.

### rABRa-A is Released from F1G4-IT by Trx-TrxR System

Our results revealed that the pathway of internalization and trafficking of F1G4-IT was distinct from that of abrin. This raised the question as to how is the A chain released from the conjugate, as it is known that the catalytic activity of abrin is effected only when the A chain is free [5], [20]. The cytosol of eukaryotic cells is highly reductive and this reductive environment is maintained by the thioredoxin and the glutaredoxin systems [45]. It has been reported that immunoconjugates of ricin were cleaved by the thioredoxin system in cells [45]. Hence, we carried out studies to see whether the thioredoxin (Trx)-thioredoxin reductase (TrxR) is involved in the cleavage of the disulfide bond between the crosslinker, SMPT, and rABRa-A in F1G4-IT.

**Figure 6:**
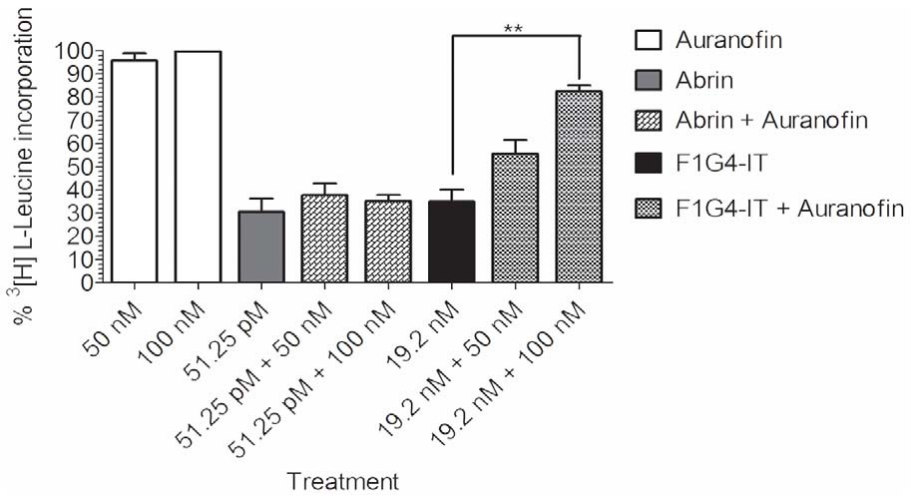
TrxR inhibitor rescues cells from F1G4-IT activity. HepG2 cells (1×10^6^/ml) were treated with auranofin for 6 h and cultured in presence of IC_90_ of either abrin (51.25 pM) or F1G4-IT (19.2 nM) for 6 h in RPMI minus leucine. The cells were then pulsed with ^3^[H] L-leucine for 2 h, total protein precipitated with 5% TCA, solubilized with 0.1 N NaOH containing 1% SDS and the incorporated radioactivity measured. The percent radioactivity above control was determined. The graph depicts the mean of at least three different experiments carried out with duplicate samples. **P<0.05.

We assayed for the inhibition of protein synthesis by the IT in HepG2 cells in the presence and absence of auranofin, a selective TrxR inhibitor [45]. Our observations revealed that the immunoconjugate was indeed reduced by the Trx-TrxR system as the inhibition of protein synthesis observed in HepG2 cells, was rescued by the inhibitor in a dose dependent manner (Fig. 6). The fact that there was no rescue of abrin activity by the inhibitor indicated that the two proteins had distinct pathways of trafficking within the cell and that abrin was reduced in the ER, with no involvement of the Trx-TrxR system in the cytosol.

## Discussion

Immunotoxins, as reported so far, have targeted mostly hematologic tumors. The available literature on studies targeting solid tumors has shown that though difficult, solid tumors can be controlled successfully using these reagents in combination with chemotherapy [53]–[56].

Abrin is a potent candidate RIP which, like other type II RIPs, inhibits protein synthesis in eukaryotes [5], [20]. The toxin also induces cell death [44], [46], [50]. In our study, we utilized abrin to construct immunotoxins to target adherent cells and chose the GnRH-R as the target molecule. Among the various surface proteins up-regulated in tumors of the pituitary, ovary and breast, two molecules are GnRH-R and EpCAM [28], [57]. In adults, GnRH-R is mainly confined to the pituitary, with very low expression in other tissues like ovary, breast, placenta etc. [24]–[26], [58]. In case of carcinomas of these tissues, the receptor levels increase significantly [24], [30], [59], [60], making it an appropriate target for therapeutic purposes. Even certain hepatocellular carcinomas like HepG2 have been reported to express the receptor whereas their normal counterparts do not express the protein [61], [62]. Hence, we targeted GnRH-R on breast and liver carcinoma cells.

Having confirmed the specificity of the antibodies to the receptors, and obtaining active recombinant abrin-a A chain, the immunotoxins were constructed using conventional chemical conjugation methods [63], [64]. The antibodies were conjugated to rABRa-A, and then analyzed for their activities on cell lines. Our observations revealed that the F1G4-IT inhibited protein synthesis specifically in GnRH-R positive MCF-7 and HepG2 cells but not in KB cells, which lack GnRH-R. Normal breast cells, MCF-10A were also mildly sensitive to F1G4-IT as they do express low levels of GnRH-R (Fig. S1, panel D). Thus the inhibitory activity was cell-specific, unlike abrin, which inhibited protein synthesis in all the cell lines. To prove that it was rABRa-A that was the inhibitory molecule, we designed a conjugate F1G4-IT**_R167L_**, with the active site mutant of rABRa-A, wherein arginine at position 167 is mutated to leucine, leading to a 625 fold decrease in the activity of the enzyme [40]. As expected, this conjugate did not inhibit protein synthesis in cells.

Abrin induces apoptosis in cells and it does so via the intrinsic mitochondrial pathway [44], [51]. Although work has been done in elucidating the pathway connecting ribotoxic stress and apoptosis in case of RIPs like ricin and shiga toxin, not much is known about the link between protein synthesis inhibitory and apoptotic activity of abrin. Our data reveals that the induction of cell death by abrin-a A chain is independent of inhibition of protein synthesis in case of HepG2 cells, as the F1G4-IT**_R167L_** which failed to inhibit protein synthesis, was able to induce cell death and the extent of cell death observed was similar to that seen with F1G4-IT. Studies are currently on to identify whether this is a general phenomenon or not and also which domain of abrin A chain is associated with the apoptotic activity.

Although both abrin and F1G4-IT inhibited protein synthesis completely by 8 h, the initial kinetics of inhibition was different. To analyze this, we carried out confocal microscopy and sub-cellular fractionation of HepG2 cells to determine the intracellular trafficking of the two proteins. Abrin, as other type II RIPs, was expected to traverse through the well-established retrograde pathway [33]. However, we made a novel finding for abrin in case of HepG2 cells. Two hours after treatment, the abrin A chain was seen to localize in the nucleus. A similar phenomenon was observed also in KB cells, and this localization is probably aided by the interaction of the A chain with a protein of ∼23 kDa, which is present in these cells, but not in cells like OVCAR-3, where we do not observe nuclear localization of the A chain of abrin (unpublished observations). These observations lead to the hypothesis that after the release into the cytosol, the A chain is sequestered into the nucleus, probably as a defence mechanism, to overcome the stress caused by toxins wherein the nucleus is acting as a ‘sink’ for proteins, similar to the sequestration of viral proteins [65]. Studies are underway to identify the interacting protein as well as the reason for nuclear localization of the A chain of abrin in certain cells.

The F1G4-IT, on the other hand, localized to the cytoplasm of HepG2 cells, with no transport to the ER or the nucleus (Fig. 4, panel B & Fig. 5, panel B). This pointed to a different and a distinct pathway of internalization and trafficking of the immunoconjugate from that of abrin. The rate of internalization of abrin and its IT also appeared different, which can be attributed to the fact that the internalization of receptors to which abrin binds could be faster and their number would also be higher as compared to the levels of GnRH-R and its rate of internalization. The trafficking of GnRH-R, when bound to agonists is well-established [66], [67]. The receptor is internalized via either coated or uncoated pits, with a rate of 30–35% in 2–3 h. Once internalized, the receptors are either recycled back to the surface of the cells, or they are targeted to lysosomes for degradation. In the interim period, the ligand bound to the receptor brings about the signalling cascade. We hypothesize that before either of the two events, receptor recycling or receptor degradation occur, the immunoconjugate bound to the receptor is released into the cytosol. However the mechanism of release of the IT from the endosomes to the cytosol is not known presently.

**Figure 7:**
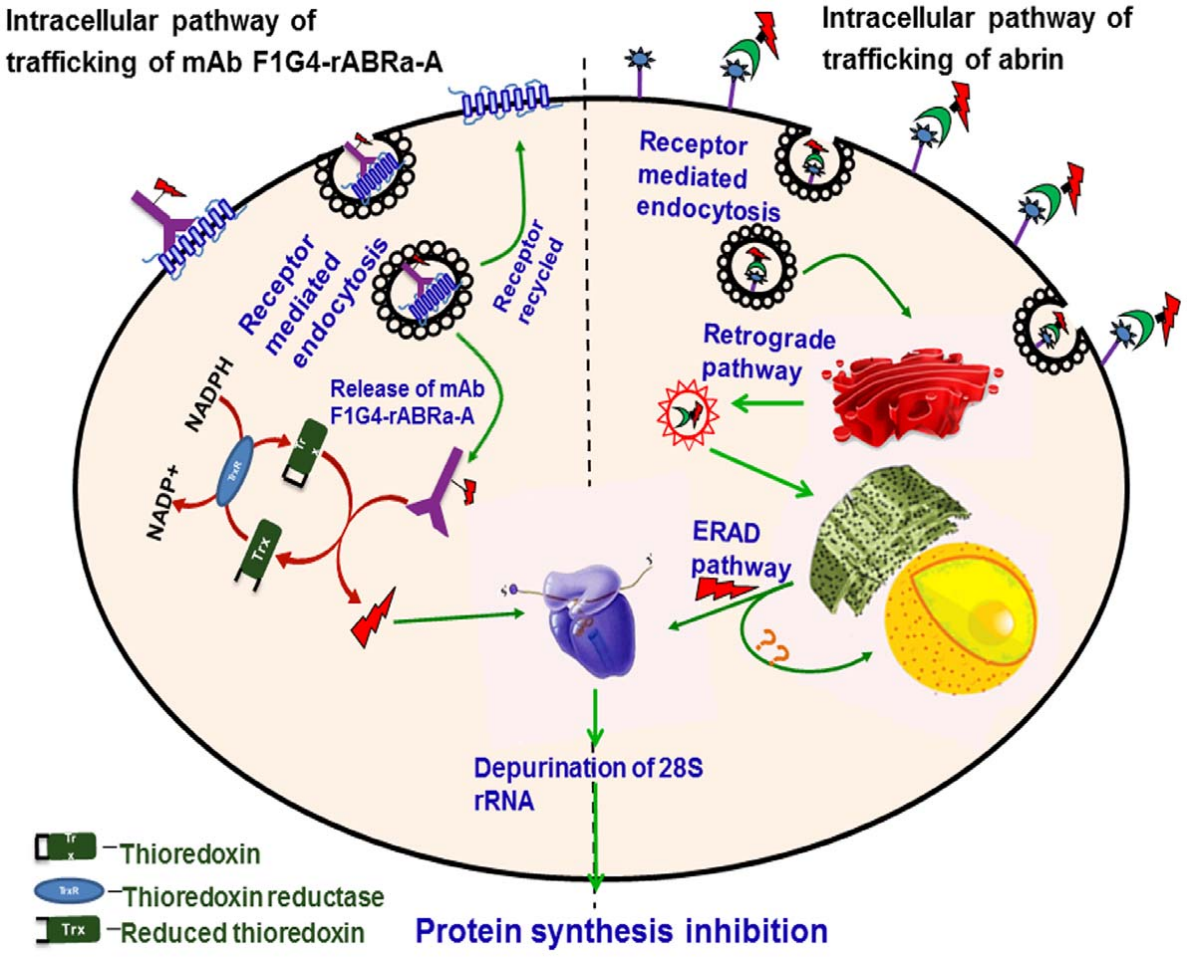
Novel intracellular trafficking of abrin and its IT. F1G4-IT binds to the GnRH receptor via the antibody, mAb F1G4, and internalized via receptor-mediated endocytosis through clathrin coated pits. The protein is then released from the vesicles into the cytosol where the S-S bond between rABRa-A and the cross-linker SMPT is cleaved by thioredoxin, releasing the recombinant A chain. The thioredoxin, on the other hand, gets oxidized which is reduced back by the enzyme, thioredoxin reductase, using protons donated by cytosolic NADPH. This pathway is different from that observed for abrin, shown in the right half of the figure, wherein the internalized protein follows the retrograde pathway to reach the ER. In the ER, the disulfide bond is cleaved, releasing the A chain to the cytosol through the ERAD pathway. Once in the cytosol, irrespective of the pathway followed, the A chain binds to the 60S ribosomal subunit, depurinating the 28S rRNA, thus inhibiting protein synthesis.

It was also important to understand how the A chain, bound to the antibody through an S-S bond with the cross-linker, is released to bring about inhibition of protein synthesis. Recent reports on ricin and its immunotoxin have revealed the involvement of thioredoxin-thioredoxin reductase system in the cytosol in releasing the A chain of the immunoconjugate [45]. We analyzed whether F1G4-IT also follows the same pathway by utilizing a selective inhibitor of TrxR, auranofin, a gold containing compound [68]. Our observations revealed that the inhibitor was able to rescue cells from inhibition of protein synthesis by F1G4-IT, but not by abrin. Thus, these results delineate a pathway for the IT distinct from that of abrin. Fig. 7 depicts a pictorial representation of the pathway of internalization and trafficking of abrin and F1G4-IT as we understand at present. Further studies on how the abrin A chain is sequestered into the nucleus and also how the immunoconjugate is released from the GnRH-R will provide clarity on the pathway.

In summary, this is one of the first reports on the nuclear localization of abrin, a type II RIP. Also, the immunotoxin mAb F1G4-rABRa-A, generated in our laboratory, inhibits protein protein synthesis specifically on cells expressing the GnRH receptor and the pathway of internalization of the protein is distinct from that seen for abrin.

## Supporting Information

Figure S1 **Fluorescence microscopy of MCF-7, HepG2, KB and MCF-10A cells for binding of mAbs F1G4, A9E4 and VU1D9.** Cells (0.4×10^4^/mm^2^) were fixed with paraformaldehyde and incubated with the antibodies overnight at 4°C, washed and stained with FITC-conjugated anti-mouse Ig. Prior to imaging, the cells were stained with Hoechst 33342 to stain the nucleus. **A:** Images of MCF-7 cells captured in the Olympus DSU microscope using a water immersion lens at 63× and analyzed using Image J Image Browser. **B:** HepG2 cells captured using the Apotome.2 microscope using an oil immersion lens of 63× and analyzed with AxioVision Rel 4.8.2. **C:** Images of KB cells captured in the Olympus DSU microscope. **D**: Images of MCF-10A captured using the Apotome.2 microscope.
(TIF)

Figure S2 **rABRa-A expressed in** ***E. coli*** **is functionally active, enabling the construction of the ITs. A:** rABRa-A and rABRa-A (R167L) were expressed in *E. coli* and purified using Ni-NTA chromatography. The purity of the proteins was determined by SDS-PAGE followed by Coomassie blue staining. **a:** rABRa-A; **b:** rABRa-A (R167L). **B:** The purified recombinant proteins were analyzed for their translation inhibitory activity using the *in vitro* translation assay. Here, rabbit reticulocyte lysate was treated with different concentrations (10 pM to 1 nM) of rABRa-A or rABRa-A (R167L) in a cocktail containing luciferase mRNA. The extent of luciferase synthesized by the lysate, in presence of the protein, was analyzed by adding luciferase substrate and determining the extent of luminescence produced. **C:** Construction and purification of immunotoxin: MAb F1G4 was conjugated to rABRa-A using SMPT as the crosslinker. **a:** The conjugate, purified on Cibacron blue 3GA affinity column was tested for purity on a 7.5% polyacrylamide SDS-gel under non-reducing conditions and immunoblotted with mAb D6F10-biotin. Lanes: 1∶5 µg mAb F1G4-rABRa-A; 2∶5 µg rABRa-A; 3∶1 µg mAb F1G4. **b:** The purified conjugate, obtained from Cibacron blue column, was re-purified using protein A affinity column to remove any remaining free A chain. The purity of the samples was tested on a 7.5% polyacrylamide SDS gel under non-reducing conditions and immunoblotted with mAb D6F10. Lanes: 1: Load; 2: Flow through; 3−4: Washes; 5−7: elution fractions.
(TIF)

Figure S3 **MCF-7 cells are more sensitive than MCF-10A to mAb F1G4-rABRa-A induced toxicity.** MCF-7 and MCF-10A cells (1×10^6^/ml) were cultured in the presence of different concentrations of F1G4-IT and assayed for protein synthesis as described earlier. The incorporated radioactivity for each sample was plotted as % of that for the control cells. Each lane represents a mean of at least three different experiments, with each treatment carried out in duplicates.
(TIF)

Figure S4 **FACScan profiles of HepG2 cells treated with abrin, F1G4-IT or F1G4-IT_(R167L)_.** HepG2 cells (1×10^6^/ml) were treated with 19.2 nM of either one of the immunoconjugates: F1G4-IT or F1G4-IT**_R167L_**, or abrin (51.25 pM) for different time intervals. The cells were harvested, fixed with 70% ethanol at −20°C, stained with staining solution (20 µg/ml propidium iodide and 50 µg/ml RNase A in PBS) and analyzed by flow cytometry. The samples were analyzed by WinMDI v2.9. The X-axis is the mean fluorescence intensity of PI and the Y-axis, the cell number, as events. Each profile indicates the statistics of cells in sub-G0/G1 stage, as M1, which indicates the extent of DNA fragmentation, a direct correlation to cells undergoing cell death. **a:** Cells treated with abrin; **b:** Cells treated with F1G4-IT; **c:** Cells treated with F1G4-IT_(R167L)_.
(TIF)
