# Supplementary figures and images for: Abrin Immunotoxin: Targeted Cytotoxicity and Intracellular Trafficking Pathway
Source: PLoS One. 2013 Mar 5;8(3):e58304. doi: 10.1371/journal.pone.0058304 (PMC3589266; doi:10.1371/journal.pone.0058304)

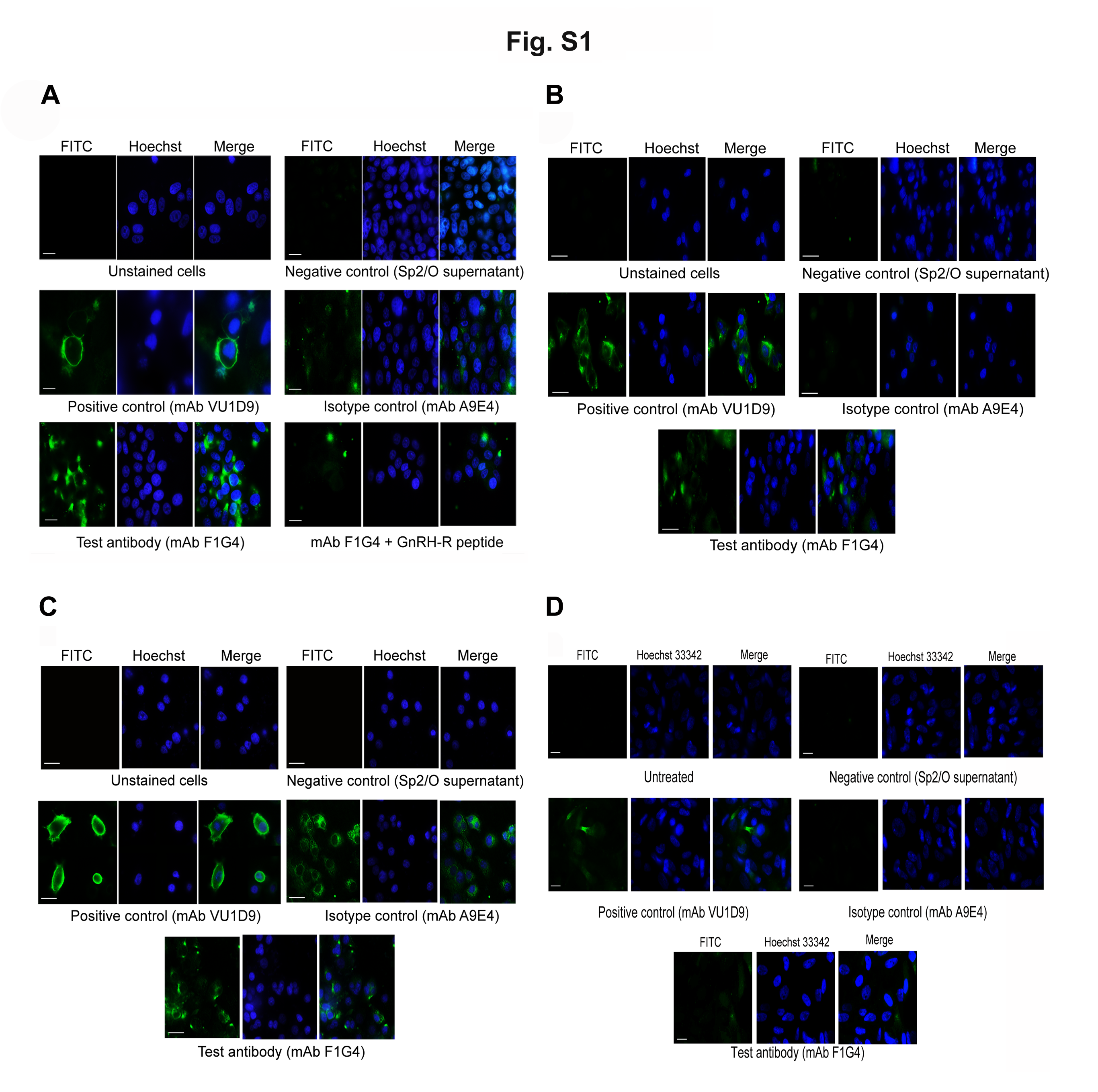

Supplement: Figure S1 — Fluorescence microscopy of MCF-7, HepG2, KB and MCF-10A cells for binding of mAbs F1G4, A9E4 and VU1D9. Cells (0.4×10^4^/mm2) were fixed with paraformaldehyde and incubated with the antibodies overnight at 4°C, washed and stained with FITC-conjugated anti-mouse Ig. Prior to imaging, the cells were stained with Hoechst 33342 to stain the nucleus. A: Images of MCF-7 cells captured in the Olympus DSU microscope using a water immersion lens at 63× and analyzed using Image J Image Browser. B: HepG2 cells captured using the Apotome.2 microscope using an oil immersion lens of 63× and analyzed with AxioVision Rel 4.8.2. C: Images of KB cells captured in the Olympus DSU microscope. D: Images of MCF-10A captured using the Apotome.2 microscope. (TIF) [file pone.0058304.s001.tif]

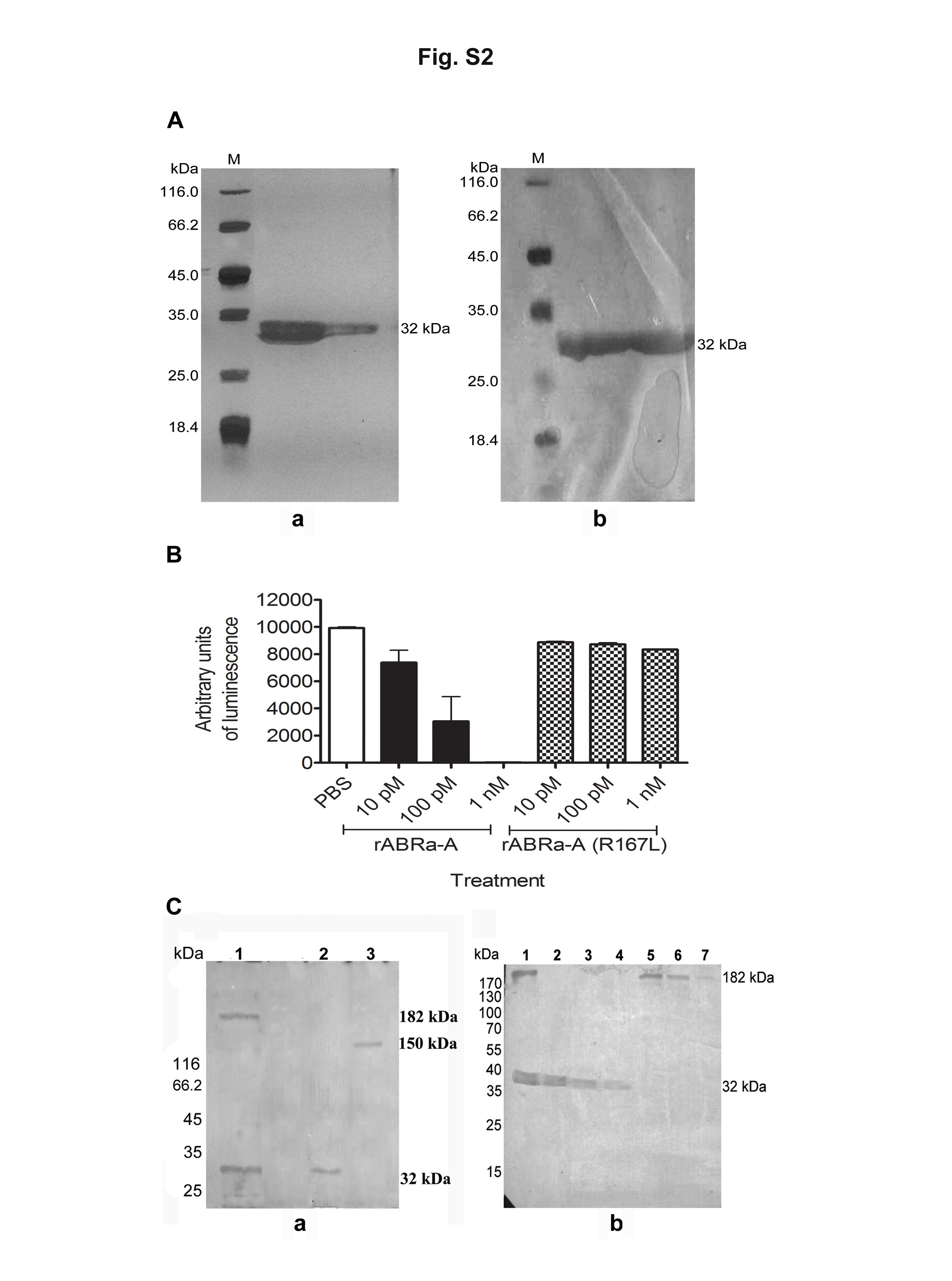

Supplement: Figure S2 — rABRa-A expressed in E. coli is functionally active, enabling the construction of the ITs. A: rABRa-A and rABRa-A (R167L) were expressed in E. coli and purified using Ni-NTA chromatography. The purity of the proteins was determined by SDS-PAGE followed by Coomassie blue staining. a: rABRa-A; b: rABRa-A (R167L). B: The purified recombinant proteins were analyzed for their translation inhibitory activity using the in vitro translation assay. Here, rabbit reticulocyte lysate was treated with different concentrations (10 pM to 1 nM) of rABRa-A or rABRa-A (R167L) in a cocktail containing luciferase mRNA. The extent of luciferase synthesized by the lysate, in presence of the protein, was analyzed by adding luciferase substrate and determining the extent of luminescence produced. C: Construction and purification of immunotoxin: MAb F1G4 was conjugated to rABRa-A using SMPT as the crosslinker. a: The conjugate, purified on Cibacron blue 3GA affinity column was tested for purity on a 7.5% polyacrylamide SDS-gel under non-reducing conditions and immunoblotted with mAb D6F10-biotin. Lanes: 1∶5 µg mAb F1G4-rABRa-A; 2∶5 µg rABRa-A; 3∶1 µg mAb F1G4. b: The purified conjugate, obtained from Cibacron blue column, was re-purified using protein A affinity column to remove any remaining free A chain. The purity of the samples was tested on a 7.5% polyacrylamide SDS gel under non-reducing conditions and immunoblotted with mAb D6F10. Lanes: 1: Load; 2: Flow through; 3−4: Washes; 5−7: elution fractions. (TIF) [file pone.0058304.s002.tif]

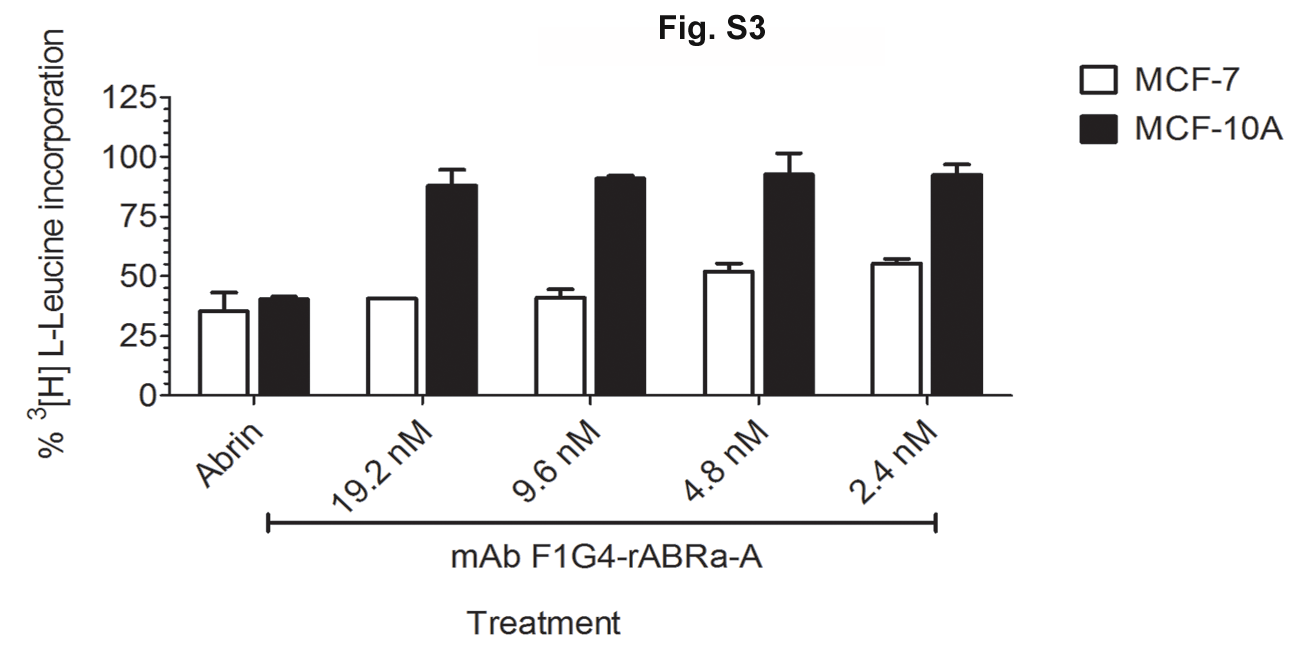

Supplement: Figure S3 — MCF-7 cells are more sensitive than MCF-10A to mAb F1G4-rABRa-A induced toxicity. MCF-7 and MCF-10A cells (1×10^6^/ml) were cultured in the presence of different concentrations of F1G4-IT and assayed for protein synthesis as described earlier. The incorporated radioactivity for each sample was plotted as % of that for the control cells. Each lane represents a mean of at least three different experiments, with each treatment carried out in duplicates. (TIF) [file pone.0058304.s003.tif]

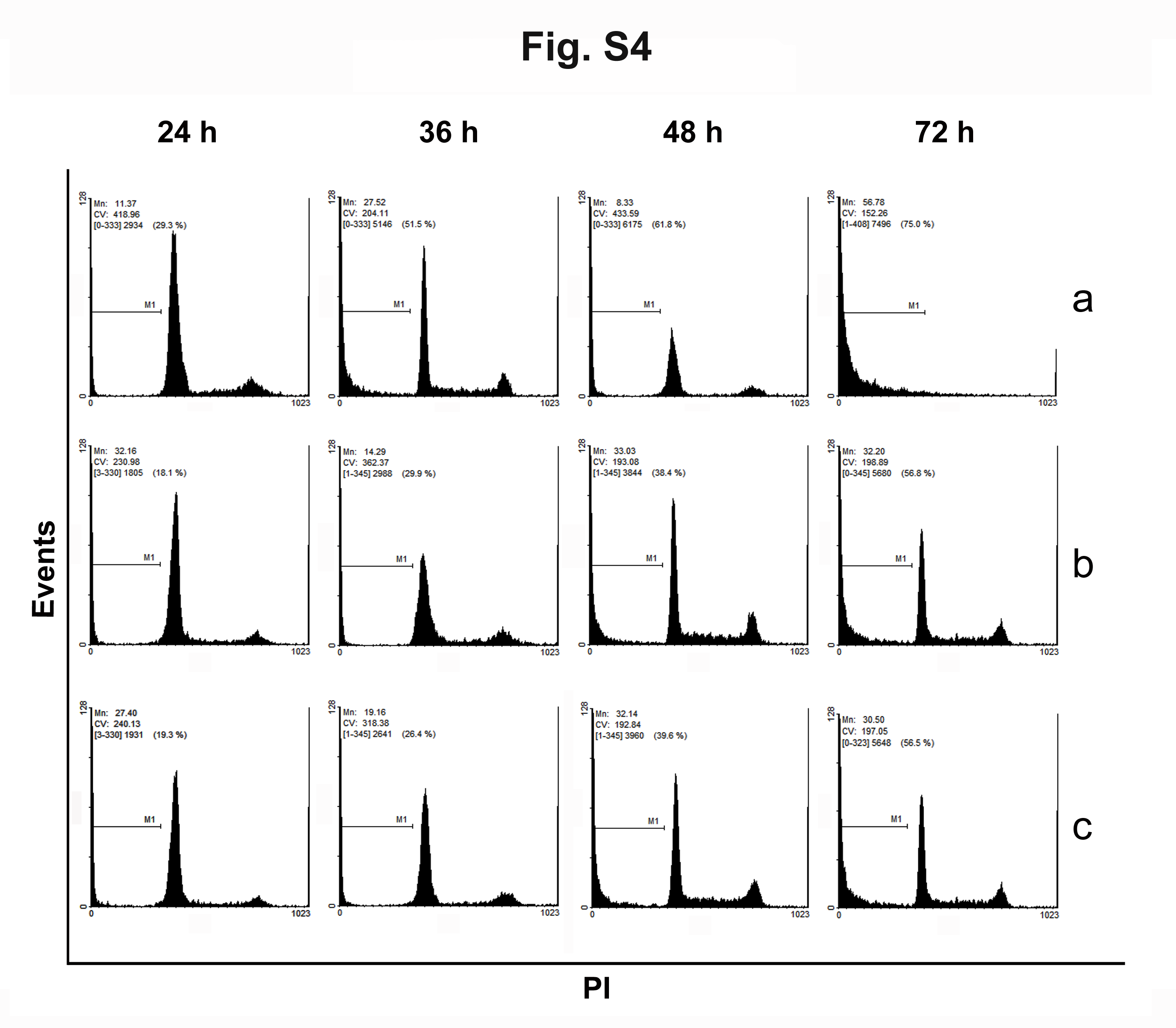

Supplement: Figure S4 — FACScan profiles of HepG2 cells treated with abrin, F1G4-IT or F1G4-IT(R167L). HepG2 cells (1×10^6^/ml) were treated with 19.2 nM of either one of the immunoconjugates: F1G4-IT or F1G4-ITR167L, or abrin (51.25 pM) for different time intervals. The cells were harvested, fixed with 70% ethanol at −20°C, stained with staining solution (20 µg/ml propidium iodide and 50 µg/ml RNase A in PBS) and analyzed by flow cytometry. The samples were analyzed by WinMDI v2.9. The X-axis is the mean fluorescence intensity of PI and the Y-axis, the cell number, as events. Each profile indicates the statistics of cells in sub-G0/G1 stage, as M1, which indicates the extent of DNA fragmentation, a direct correlation to cells undergoing cell death. a: Cells treated with abrin; b: Cells treated with F1G4-IT; c: Cells treated with F1G4-IT(R167L). (TIF) [file pone.0058304.s004.tif]
